# Featured Organism: Reductive Evolution in Bacteria: *Buchnera* sp., *Rickettsia Prowazekii* and *Mycobacterium Leprae*

**DOI:** 10.1002/cfg.70

**Published:** 2001-02

**Authors:** Jo Wixon

**Affiliations:** Bioinformatics Division HGMP-RC Hinxton, Cambridge CB10 1SA UK

## Abstract

Obligate intracellular bacteria commonly have much reduced genome sizes compared to their nearest free-living relatives. One reason for this is reductive evolution: the loss of genes rendered non-essential due to the intracellular habitat. This can occur because of the presence of orthologous genes in the host, combined with the ability of the bacteria to import the protein or metabolite products of the host genes. In this article we take a look at three such bacteria whose genomes have been fully sequenced. *Buchnera* is an endosymbiont of the pea aphid, *Acyrthosiphon pisum*, the relationship between these two organisms being so essential that neither can reproduce in the absence of the other. *Rickettsia prowazekii* is the causative agent of louse-borne typhus in humans and *Mycobacterium leprae* infection of humans leads to leprosy. Both of these human pathogens have fastidious growth requirements, which has made them very difficult to study.


**Comparative and Functional Genomics**
*Comp Funct Genom* 2001; **2**: 44–48.

**Featured Organism**

### Reductive evolution in bacteria: *Buchnera* sp., *Rickettsia prowazekii* and *Mycobacterium leprae*

Jo Wixon, Managing Editor
*J. Wixon, Bioinformatics Division, HGMP-RC, Hinxton, Cambridge CB10 1SA, UK*

### Abstract

**Obligate intracellular bacteria commonly have much reduced genome sizes compared to their nearest free-living relatives. One reason for this is reductive evolution: the loss of genes rendered non-essential due to the intracellular habitat. This can occur because of the presence of orthologous genes in the host, combined with the ability of the bacteria to import the protein or metabolite products of the host genes. In this article we take a look at three such bacteria whose genomes have been fully sequenced. *Buchnera* is an endosymbiont of the pea aphid, *Acyrthosiphon pisum*, the relationship between these two organisms being so essential that neither can reproduce in the absence of the other. *Rickettsia prowazekii* is the causative agent of louse-borne typhus in humans and *Mycobacterium leprae* infection of humans leads to leprosy. Both of these human pathogens have fastidious growth requirements, which has made them very difficult to study.** **Copyright © 2001 John Wiley & Sons, Ltd.**

### Background

### *Buchnera* sp.

*Buchnera* is a close relative of *Escherichia coli* and *Haemophilus influenzae*. It exists at around 100 copies per cell in specialized cells (bacteriocytes) found within almost all aphid species. These cells are transmitted from the mother via the eggs and thus the bacterium is passed on to following generations of the fly. The complete sequence of the genome of *Buchnera* sp. APS (which is roughly one-seventh of the size of that of *E. coli*) was published in September 2000 (Shigenobu *et al.*, 2000). The 640 681 bp genome was found to contain 583 ORFs and is AT-rich, with a G+C content of just 26.3%. Of the 583 ORFs, 500 were assigned a functional category, whilst 79 were conserved hypotheticals and just four were unique to *Buchnera*. The majority of *Buchnera* genes were found to have closest relatives in *E. coli*, which led to the conclusion that its genome is essentially a subset of that of *E. coli*.

### *Rickettsia prowazekii*

Carried by lice, upon entry into humans *R. prowazekii* causes epidemic typhus, which has killed in the region of 30 million people since the First World War. To date, this $\alpha$-proteobacterium is more closely related to the mitochondrion than any other microbe studied. Its genome was fully sequenced and published in November 1998 (Andersson *et al.*, 1998); 834 protein-coding genes were identified, representing just 75.4% of the 1 111 523 base pairs (bp) of the chromosome. A function was assigned to 62.7% of these genes, while 12.5% were conserved hypotheticals and 24.8% had no similarity to genes found in any other sequenced organism. 24% of the genome is non-coding, only a small fraction of the genome (0.9%) corresponds to pseudogenes and even less (0.2% of the genome) consists of non-coding repeats. So, the majority of this non-coding material is made up of regions of lower G+C content (on average 23.7%) characteristic of *R. prowazekii* intergenic (spacer) sequence.

Copyright © 2001 John Wiley & Sons, Ltd.

### *Mycobacterium leprae*

*M. leprae* is the causative agent of leprosy in humans. It is most closely related to *M. tuberculosis* but, again, it has a much reduced genome size. At 3 268 182 bp, its genome is around 1.2 Mb smaller that that of its relative. This difference is partly due to the presence of a higher proportion of repetitive DNA in the *M. tuberculosis* genome. The *M. leprae* genome has a very much lower GC content, at 58%, compared to 66%, and a much lower proportion is coding sequence, ~50% compared to 90%. Of the genes identified from the sequence, 1501 have *M. tuberculosis* homologues, ~100 are unique to *M. leprae* and 782 are pseudogenes. There are ~1700 *M. tuberculosis* genes with no *M. leprae* orthologue. Sixty-five segments of conserved gene order have been identified between *M. leprae* and *M. tuberculosis*, implying that there have been a large number of rearrangements, most likely caused by recombinations between repetitive elements.

### What is reductive evolution?

In intracellular bacteria, it is possible for genes to be rendered inessential due to the activity of homologous host genes. This then removes the selective pressure against the occurrence of mutations within such a gene. The accumulation of mutations can result in the inactivation of a particular gene. The intracellular environment can act as an evolutionary bottleneck, by preventing the recovery of such mutations by exchange of material with other bacteria. This can then fix these mutations in the population. The final step of the process can be the deletion of these genes (see Andersson and Kurland, 1998, for a review).

The process of loss of fitness in a population is referred to as 'Muller's ratchet' (Felsenstein, 1974; Muller, 1964). The loss of the affected genes, which are essential for free-living organisms, causes the organism to adopt an obligate intracellular lifestyle. This process can occur to the genes of both parasitic and symbiotic intracellular organisms, as our examples illustrate. However, whereas endosymbionts never leave their hosts, the most extreme example of this being the mitochondrion, obligate intracellular parasites can often need to find a new host and may be adapted to survival within another organism.

The accumulation of mutations in non-essential genes is, however, not enough to result in a smaller genome, and a further part of the process is reduction in genome size by deletion. It appears that the principal mechanism for deletion (or insertion) in bacterial genomes is recombination between repeated sequences on the chromosome (Krawiec and Riley, 1990). These events can rearrange parts of the genome (when the repeats are in inverse orientation) or result in the deletion of the material between the repeats (if they are in the same orientation). Unlike the situation for free-living bacteria, in which these large-scale variations are rarely observed, such events seem to occur at a significantly higher rate in intracellular bacteria, most likely causing the smaller, rearranged genomes that we are now discovering. Even genomic regions that show very high conservation of gene order in free-living species appear to be rearranged in obligate intracellular species. In fact, small genome size and scrambled gene order seem to be characteristics of these species.

### Which genes have been lost?

Genes involved in the biosynthesis of nutrients are those most commonly lost amongst bacteria showing reductive evolution. This is clearly the case in *Rickettsia*, which has a very small proportion of biosynthetic genes. For example, it has no genes for *de novo* nucleotide synthesis, only those for conversion of nucleoside monophosphates into all other nucleotides, implying that nucleoside monophosphates are taken up from the host. This story is the same in *Chlamydia* (Zomorodipour and Andersson, 1999), which has no common intracellular ancestor with *Rickettsia*, implying that this has resulted from convergent reductive evolution.

*Rickettsia* has a very small number of genes involved in amino acid biosynthesis, all of which have other roles that could explain why they have been retained. It has glyA (which has a role in tetrahydrofolate metabolism), seven enzymes which perform just the first few steps of lysine, methionine and threonine biosynthesis (producing diaminopimelate, an essential cell envelope component), and other genes which have a limited role in amino acid biosynthesis, but which also seem to have deamination roles, diverting amino acids into the tricarboxylic acid cycle.

While it is known to take up ATP from the host

Copyright © 2001 John Wiley & Sons, Ltd.

in the early stages of infection, and has genes for ATP/ADP translocases, *Rickettsia* can produce ATP, using the TCA cycle and respiratory-chain complexes. However, it does not have the genes required for anaerobic glycolysis. There are also genes for the three components of the pyruvate dehydrogenase complex, implying that, like mitochondria, *Rickettsia* imports cytosolic (host) pyruvate.

*Rickettsia* has fewer genes involved in replication than its free-living relatives. It has the four genes that encode the core components of DNA polymerase III, but not those for the extra subunits present in the *E. coli* complex. It has the genes required for repair of UV-induced damage, and also the genes for an excision repair pathway like that in *Borrelia burgdorferi*, but it has only a limited capacity for mismatch repair, having just the mutL and mutS genes. It has several of the genes for homologous recombination, but the recBCD complex is absent.

When compared to *M. tuberculosis*, the genes that *M. leprae* has lost can be seen to have had catabolic functions. It has no P450 genes, an almost completely deleted NADH oxidase operon and no siderophores. An example where genes have become pseudogenes is seen in those genes involved in the pathway used to modify mycolic acids into the $\alpha$-, keto- and methoxy-forms (Brosch *et al.*, 2000). In *M. tuberculosis* there are four clustered genes: mmaA1, A2, A3 and A4. In the syntenic region of the *M. leprae* genome, only functional copies of mmaA1 and A4 remain, and the mmaA2 and A3 genes are present as pseudogenes. This could explain the lack of methoxy-mycolates in the *M. leprae* cell wall, whereas they are a component of the wall of the tubercle bacillus.

*M. leprae* is recombination-deficient; it has no dnaQ. It has no recA and no recBCD complex, which could mean that it is also deficient in repair by homologous recombination.

The pseudogenes that are found in *M. leprae* are inactivated versions of genes that are still functional in *M. tuberculosis*. These are most likely the remains of genes whose functions have been rendered inessential, e.g. by the uptake of compounds produced by the host. The natural selection against mutation in these genes will therefore have been lost.

The genome of *M. leprae* is scrambled compared to that of *M. tuberculosis*. This has most likely been caused by recombinations between repeats. It also shows signs of downsizing and decay (Cole, 1998). Its naturally selected (potentially) minimal mycobacterial gene set could be taken to imply that it may be a much older pathogen than *M. tuberculosis*.

Unlike these two pathogens, *Buchnera* has retained certain biosynthetic pathways, which supply products that the aphid host cannot make. The pattern of gene presence is symbiotic, in that the genes that have been lost in *Buchnera* are for the synthesis of non-essential amino acids (which are supplied by the aphid), whilst it supplies essential amino acids and vitamins to the host (Shigenobu *et al.*, 2000). Some of the pathways are mutually dependent, e.g. the aphids recycle amino groups as glutamine (rather than excreting nitrogenous waste), which is then used by *Buchnera* in the synthesis of essential amino acids (for a review of such interactions, see Douglas, 1998). These complementary patterns of gene presence are the result of a very old association between the bacterium and the fly, phylogenetic analyses suggest that the symbiotic relationship between them is 200–250 million years old.

In contrast to the parasites described above, *Buchnera* has almost complete nucleotide biosynthetic pathways. It can synthesize histidine, phenylalanine and tryptophan (and, it is thought, valine and leucine) independently of the aphid, but is believed to use precursors supplied by the aphid to produce threonine, methionine, lysine, arginine and isoleucine. The majority of the genes for synthesizing non-essential amino acids such as glutamate and glutamine are absent.

*Buchnera* has complete gene complements for glycolysis and the pentose phosphate cycle. It appears to respire aerobically, taking advantage of the aerobic environment provided by the bacteriocyte, and has lost the genes for anaerobic respiration and fermentation. It does not have all the genes required to utilize the TCA cycle and is apparently unable to synthesize ubiquinone. It does, however, have an operon that encodes an $F_0F_1$ type ATP synthase, which uses the proton electrochemical gradient made by the electron transport system to produce ATP.

*Buchnera* appears to be somewhat deficient in DNA repair; unusually, it has no recA gene but still has the recBCD operon and also has incomplete gene complements for the *uvr* excision repair and SOS systems. It also appears to have a less structurally sound cell surface than many free-living bacteria, since it is unable to make lipopolysaccharides. The absence of genes for making

Copyright © 2001 John Wiley & Sons, Ltd. 

phospholipids was a surprise; it seems that *Buchnera* must either make them using imported host enzymes or else it constructs its membrane lipid bilayer using host phospholipid. This level of dependence on the host is far greater than that of the parasites discussed above.

The nature of the relationship between *Buchnera* and the aphid leads to the expectation that it will have a wide selection of transport genes to facilitate the transfer of nutrients to and from the host cytoplasm. However, this does not appear to be the case; the only substrate-specific transporters in *Buchnera* are a few ABC transporter genes and phosphotransferase systems (PTSs) for glucose and mannitol. The group that sequenced the genome suggest that the flagellum might act as a transporter structure (Shigenobu *et al.*, 2000) as in some other bacteria, since the gene for the filament (which gives motility) is absent.

### Other signs of reductive evolution

The first indicator that the deletion of large regions of these genomes has occurred is their small size compared to their free-living relatives. The *Buchnera* genome is roughly one-seventh of the size of that of *E. coli* and the genome of *M. leprae* is more than 1 Mb smaller than the *M. tuberculosis* genome. The *Rickettsia* genome is also very small, at just 1 Mb.

Comparison of gene content with their free-living relatives shows just how many genes may have been lost, *M. tuberculosis* has almost 2000 genes for which there is no *M. leprae* homologue and the *Buchnera* gene set appears to be just a subset of that of *E. coli*.

More detailed comparisons show that many rearrangements have occurred in these genomes. Despite their close phylogenetic relationship, conserved gene order between the genomes of *M. tuberculosis* and *M. leprae* now exists as 65 separate regions. *Rickettsia* shows rearrangement of regions in which gene order is highly conserved in the genomes of its free-living relatives; even the ribosomal protein gene operons and rRNA genes have been subjected to inversion and deletion events (Andersson *et al.*, 1999; Syvänen *et al.*, 1996). This does not appear to be so much the case in *Buchnera*, which shows good conservation of gene order in operons, compared to *E. coli*.

One gene in particular, that encoding S-adenosylmethionine synthase (metK) in *Rickettsia*, has been shown to be in the process of being lost from the genome. This gene (along with 11 other genes) was found to be a pseudogene in the sequenced *R. prowazekii* genome (Andersson *et al.*, 1998), containing a termination codon in what is normally a highly conserved region of the gene. Further study of this gene and neighbouring genes showed that metK, but not its neighbours, contained mutations in six out of eight species of the *Rickettsia* genus (Andersson and Andersson, 1999). More detailed examination of the nature of the mutations showed that they were predominantly deletions and that there was an excess of GC to AT substitutions amongst the point mutations. This could explain the low overall G+C content in the *Rickettsia* genome (on average 29%).

### Web-based resources

Integrated *Buchnera* Genome Database (iBGD)
**http://buchnera.gsc.riken.go.jp/**

The Department of Molecular Evolution, Uppsala University
(research on the evolution of *Rickettsia*, mitochondria and *Buchnera*)
**http://web1.ebc.uu.se/molev/**

TIGR Comprehensive Microbial Resource
(*R. prowazekii*, *Chlamydia*, *E. coli* and *M. tuberculosis* data)
**http://www.tigr.org/tigr-scripts/CMR2/CMRHomePage.spl**

The Sanger Centre *M. leprae* project
**http://www.sanger.ac.uk/Projects/M_leprae/**

Mycobacterial genomics
(*M. tuberculosis*, *M. bovis* and *M. leprae* genomics)
**http://www.pasteur.fr/recherche/unites/Lgmb/mycogenomics.html**

*M. leprae cosmid sequences from Genome Therapeutics Corporation*
**http://www.genomecorp.com/genesequences/index.html**

Indo-Swiss collaboration in biotechnology *M. leprae* project
**http://ibtech.ethz.ch/india/project4.html**

WHO information on leprosy
**http://www.who.int/lep/**

Copyright © 2001 John Wiley & Sons, Ltd.

### References

Andersson JO, Andersson SG. 1999. Genome degradation is an ongoing process in *Rickettsia*. *Mol Biol Evol* **16**: 1178–1191.

Andersson SG, Kurland CG. 1998. Reductive evolution of resident genomes. *Trends Microbiol* **6**: 263–268.

Andersson SGE, Stothard DR, Fuerst P, Kurland CG. 1999. Molecular phylogeny and rearrangement of rRNA genes in *Rickettsia* species. *Mol Biol Evol* **16**: 987–995.

Andersson SGE, Zomorodipour A, Andersson JO, *et al*. 1998. The genome sequence of *Rickettsia prowazekii* and the origin of mitochondria. *Nature* **396**: 133–140.

Baumann P, Baumann L, Lai CY, *et al*. 1995. Genetics, physiology, and evolutionary relationships of the genus *Buchnera*: intracellular symbionts of aphids. *Ann Rev Microbiol* **49**: 55–94.

Brosch R, Gordon SV, Eiglmeier K, Garnier T, Cole ST. 2000. Comparative genomics of the leprosy and tubercle bacilli. *Res Microbiol* **151**: 135–142.

Cole ST. 1998. Comparative mycobacterial genomics. *Curr Opin Microbiol* **1**: 567–571.

Douglas AE. 1998. Nutritional interactions in insect-microbial symbioses: aphids and their symbiotic bacteria *Buchnera*. *Ann Rev Entomol* **43**: 17–37.

Felsenstein J. 1974. The evolutionary advantage of recombination. *Genetics* **78**: 737–756.

Krawiec S, Riley M. 1990. Organization of the bacterial chromosome. *Microbiol Rev* **54**: 502–598.

Muller HJ. 1964. *Mutat Res* **1**: 2–9.

Shigenobu S, Watanabe H, Hattori M, Sakaki Y, Ishikawa H. 2000. Genome sequence of the endocellular bacterial symbiont of aphids *Buchnera* sp. APS. *Nature* **407**: 81–86.

Syvänen A-C, Amiri H, Jamal A, Andersson SGE, Kurland CG. 1996. A chimeric disposition of the elongation factor genes in *Rickettsia prowazekii*. *J Bact* **178**: 6192–6199.

Zomorodipour A, Andersson SGE. 1999. Obligate intracellular parasites: *Rickettsia prowazekii* and *Chlamydia trachomatis*. *FEBS Lett* **452**: 11–15.

*Comparative and Functional Genomics* is a cross-organism journal, publishing studies on complex and model organisms. This ‘Featured Organism’ article aims to present an overview of an evolutionary mechanism common amongst particular bacterial species, primarily for those working on other systems. It provides background information and also gives a list of websites containing further information on the chosen example organisms.

Copyright © 2001 John Wiley & Sons, Ltd. 
